# The association between tea consumption and hypertension: a systematic review and meta-analysis

**DOI:** 10.3389/fnut.2026.1883708

**Published:** 2026-08-20

**Authors:** Renyu Jiang, Jinyan Chen, Hongli Lin, Qi Wang, Yong Hu

**Affiliations:** 1 First School of Clinical Medicine, Shandong University of Traditional Chinese Medicine, Jinan, China; 2 Department of Nephropathy, Qingdao Hospital of Traditional Chinese Medicine, Qingdao, China; 3 Department of Geriatrics, Qingdao Hospital of Traditional Chinese Medicine, Qingdao, China

**Keywords:** dose–response analysis, hypertension, meta-analysis, observational studies, risk factors, tea

## Abstract

**Objective:**

The association between habitual tea consumption and hypertension remains controversial. This systematic review and meta-analysis aimed to synthesize observational evidence on tea consumption and hypertension and to explore whether this association differed according to tea type, sex, age, region, and hypertension diagnostic threshold.

**Methods:**

PubMed, Embase, Cochrane Library, Web of Science, CNKI, Wanfang, and VIP were systematically searched from database inception to December 2025 for observational studies in adults. Studies reporting a distinct hypertension outcome and an extractable effect estimate for tea consumption were eligible. Study quality was assessed using the Newcastle–Ottawa Scale or AHRQ criteria, as appropriate. The most fully adjusted *ORs*, *RRs*, or *HRs* were extracted. Because these measures are not mathematically identical, pooled estimates were interpreted conservatively as relative association estimates rather than directly interchangeable causal effect measures. Dose–response analyses were conducted for studies with at least three quantitative exposure categories.

**Results:**

Twenty studies involving 319,565 participants were included. In 16 cross-sectional studies, tea consumption was weakly associated with lower hypertension prevalence (*OR* = 0.92, 95% *CI*: 0.87–0.97, *I^2^* = 62.8%). This inverse association was stronger for black tea drinkers, Asians, women, participants aged < 60 years, and studies with a hypertension cutoff of ≥140/90 mmHg; these subgroup results are hypothesis-generating, not confirmatory. Pooled *ORs* of the four cohort studies indicated a 13% lower hypertension risk (*OR* = 0.87, 95% *CI*: 0.84–0.92, *I^2^* = 31.9%). Dose–response analysis suggested a significant nonlinear association, with an initially steeper inverse pattern at lower intake levels followed by an apparent plateau at higher reported intake.

**Conclusion:**

Habitual tea consumption was associated with a modestly lower likelihood of hypertension in observational studies. Owing to the observational design and marked inter-study heterogeneity, a definite clinical protective effect cannot be confirmed.

**Systematic review registration:**

https://www.crd.york.ac.uk/PROSPERO/, CRD420261288902.

## 1 Introduction

Hypertension is a major contributor to cardiovascular morbidity and mortality and remains one of the most important preventable risk factors worldwide (1). The prevalence of hypertension among adults has reached approximately 31.1%, affecting an estimated 1.39 billion people globally (2). Despite this burden, many adults remain undiagnosed or undertreated (3, 4), and deaths attributable to hypertensive heart disease continue to rise (5). Identifying modifiable lifestyle and dietary correlates of hypertension therefore remains clinically and publicly important (6).

Tea is one of the most widely consumed beverages worldwide and contains multiple bioactive compounds, including catechins, theaflavins, caffeine, and other polyphenols (7, 8). These constituents may influence endothelial function, oxidative stress, vascular tone, and the renin–angiotensin system, thereby providing biological plausibility for an association with blood pressure regulation (8–11). However, epidemiological studies examining habitual tea consumption and hypertension have reported inconsistent findings. Accordingly, the present study was designed to synthesize observational evidence on habitual tea consumption and hypertension, to distinguish hypertension-specific outcomes from broader cardiometabolic endpoints, and to examine whether the observed association varied according to tea type, sex, age, region, diagnostic threshold, and dose.

## 2 Materials and methods

### 2.1 Terms and registration

To ensure the methodological rigor and transparency of this meta-analysis, the study was conducted in strict accordance with the MOOSE guidelines for meta-analyses of epidemiological observational studies (12) and the PRISMA Preferred Reporting Items for Systematic Reviews and Meta-Analyses (13) throughout the entire process. These guidelines provide a standardized methodological framework for the conduct and reporting of systematic reviews and meta-analyses. Furthermore, to further enhance research transparency and reduce the risk of bias, the study protocol has been registered in the international registry for systematic review protocols, PROSPERO, under registration number CRD420261288902.

### 2.2 Literature search strategies

This systematic review and meta-analysis searched PubMed, Embase, the Cochrane Library, Web of Science, CNKI, Wanfang, and VIP from database inception to December 2025 for observational studies evaluating tea consumption and hypertension. Search concepts combined controlled vocabulary, when available, and free-text terms related to tea exposure (e.g., tea, tea drinking, tea consumption, tea intake, green tea, black tea, and oolong tea) and hypertension outcomes (e.g., hypertension, high blood pressure, and elevated blood pressure). The reference lists of included studies and relevant reviews were also screened during full-text assessment to identify additional eligible records. The final search update was conducted in December 2025.

### 2.3 Inclusion criteria

To assess the association between tea intake and hypertension, eligible studies were required to meet all of the following criteria: (i) the study population comprised adults (aged ≥18 years); (ii) the study used an observational design, including prospective cohort, case–control, or cross-sectional studies; (iii) the study reported a distinct hypertension outcome or a separately extractable hypertension-specific effect estimate from a broader cardiometabolic analysis; (iv) the study provided an effect estimate for tea consumption, such as an *OR*, *RR*, or *HR*, together with a corresponding 95% confidence interval; and (v) the study allowed comparison between higher and lower categories of tea intake or provided sufficient quantitative exposure data for dose–response analysis.

### 2.4 Exclusion criteria

Studies were excluded if they met any of the following criteria: (i) the study population did not meet the inclusion criteria, including non-human studies or studies focused on clearly defined secondary hypertension; (ii) the publication was not an original research article, such as a conference abstract, case report, editorial, letter, infographic, commentary, opinion piece, position statement, or narrative review; (iii) the study did not provide extractable hypertension-specific effect estimates or sufficient quantitative information for meta-analysis, used an unclear outcome definition, or lacked key diagnostic information.

### 2.5 Literature screening and data extraction

Two authors (Jiang and Chen) independently screened titles, abstracts, and full texts according to the predefined inclusion and exclusion criteria. Disagreements were resolved through discussion and, if needed, adjudication by a third reviewer (Hu). Extracted information included first author, publication year, country, study design, tea type, tea intake assessment, hypertension definition or outcome assessment method, sample size, age, sex, effect-measure type, and the most fully adjusted effect estimate. For studies addressing broader metabolic syndrome or cardiometabolic outcomes, only hypertension-specific results that could be extracted separately were eligible for inclusion.

### 2.6 Evaluation of literature quality

Two reviewers independently assessed study quality. Cohort studies and case–control studies were evaluated using the Newcastle–Ottawa Scale (NOS) (14), and cross-sectional studies were evaluated using the Agency for Healthcare Research and Quality (AHRQ) checklist (15, 16). In addition to total scores, particular attention was paid to exposure ascertainment, outcome ascertainment, comparability or confounder adjustment, and, for cohort studies, adequacy of follow-up. Disagreements regarding study appraisal were resolved by third-reviewer adjudication.

### 2.7 Statistical analysis

All statistical analyses were performed using Stata 15.0. The most fully adjusted *ORs*, *RRs*, or *HRs* reported by the original studies were extracted whenever available. Because these relative measures are not mathematically identical, particularly for a non-rare outcome such as hypertension, pooled estimates were interpreted conservatively as relative association estimates rather than directly equivalent or causal effect measures. Cross-sectional studies and cohort studies were analyzed separately. Subgroup analyses were conducted within cross-sectional studies to explore prevalence associations. Heterogeneity was evaluated using the Cochran *Q* test and the *I^2^* statistic. When *p* > 0.1 and *I^2^* ≤ 50%, heterogeneity was considered low and a fixed-effects model was used; otherwise, a random-effects model was applied (17). Meta-regression was not retained in the final analytic plan because several prespecified strata contained relatively few studies and the mixed study designs limited interpretability; heterogeneity was instead explored using prespecified subgroup analyses and leave-one-out sensitivity analysis. Publication bias was assessed by funnel plot inspection and Egger’s test (18), and robustness was examined using stepwise exclusion (19). For dose–response analysis, the method of Greenland and Longnecker (20) was used, and restricted cubic spline models with three knots at the 10th, 50th, and 90th percentiles of the exposure distribution were fitted to evaluate potential nonlinearity (21). All exposure metrics reported as daily, weekly, or monthly tea intake were converted to cups/day when possible. When intake was expressed in grams or milliliters, conversions were made using the equivalencies of 125 g/month = 2 cups/day and 237 mL = 1 cup, respectively; this pragmatic harmonization was applied to facilitate cross-study comparability (22), and the resulting dose–response findings were interpreted cautiously. Dose assignment used the mean, median, or interval midpoint of each exposure category; open-ended categories were approximated using adjacent interval widths. A two-sided *p*-value < 0.05 was considered statistically significant.

## 3 Results

### 3.1 Literature search results

**Figure 1:**
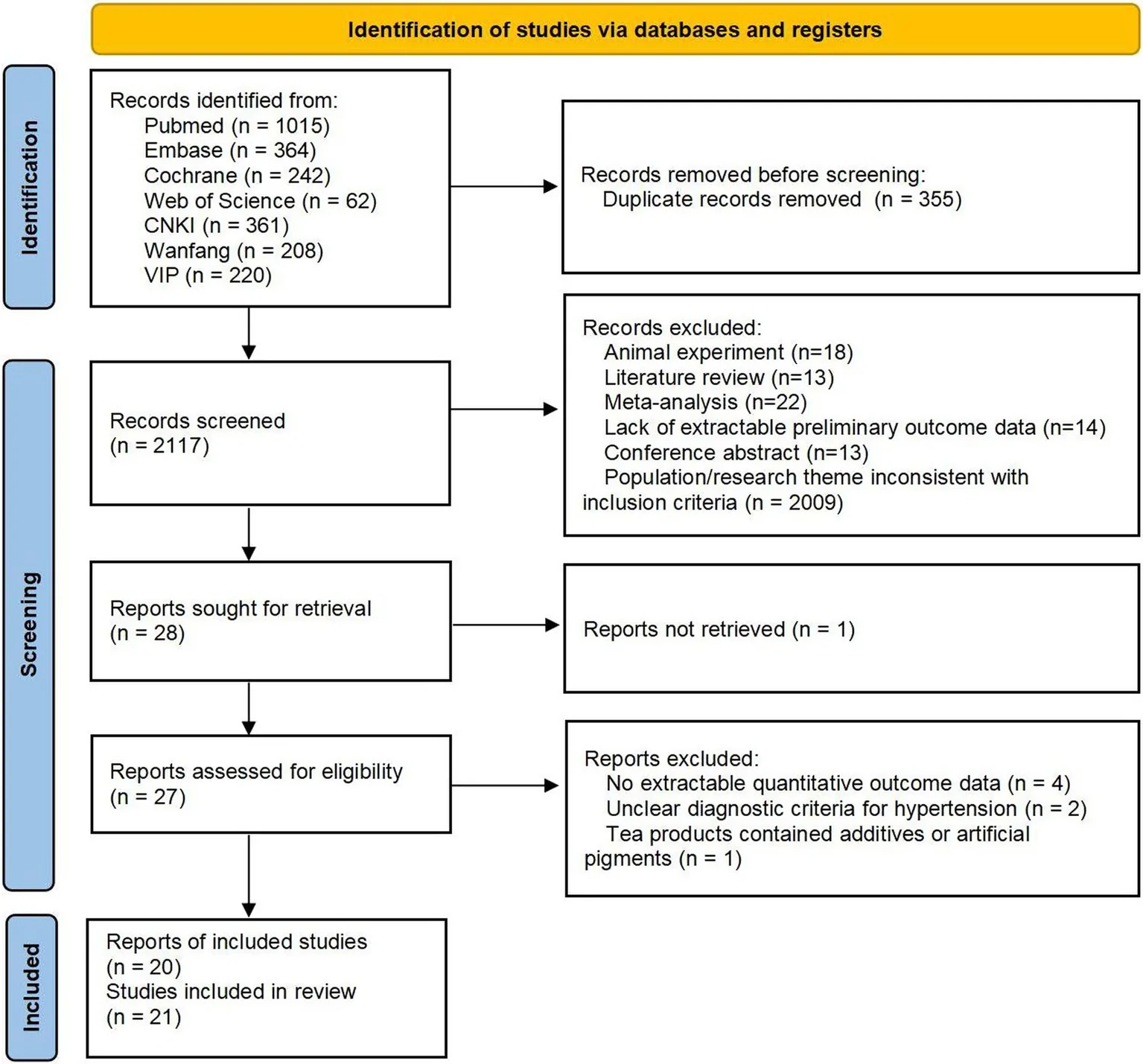
Article selection flowchart.

Figure 1 summarizes the literature screening process. A total of 2,472 records were identified through database searching, and 2,117 remained after deduplication. Titles, abstracts, and then full texts were screened according to the predefined eligibility criteria. Common reasons for full-text exclusion included non-observational design, lack of extractable hypertension-specific effect estimates, insufficient quantitative data, and unclear outcome definition. Ultimately, 20 studies were included in the meta-analysis, comprising 4 cohort studies and 16 cross-sectional studies. Although the search was updated to December 2025, no additional eligible observational studies published after 2023 met the inclusion criteria.

### 3.2 Characteristics of included studies

A total of 20 studies published between 2004 and 2023 were included in the final analysis, comprising 319,565 participants overall; 19 studies reported sex data including 128,242 men and 189,297 women, while one study lacked gender breakdowns. Geographically, 16 studies were conducted in Asia, 3 in Europe, and 1 in Africa. Regarding study design, 4 were cohort studies and 16 were cross-sectional studies. Eleven studies reported specific tea types, including green tea and black tea. Thirteen studies used systolic/diastolic blood pressure thresholds of ≥ 140/90 mmHg, seven used ≥ 130/85 mmHg. Several included reports addressed broader metabolic syndrome or cardiometabolic outcomes, but only separately extractable hypertension-specific estimates were entered into the present synthesis. Detailed study characteristics are shown in Table 1.

**Table 1:** Characteristics of the studies included in the final analysis.

Study	Country	Study design	Tea intake assessment	Tea type	Outcome assessment / HTN definition	Participants as reported	Age	Sex (male/female)
Yi-Ching Yang 2004 (34)	China	Cross-sectional study	Questionnaire	Green tea, Black tea, or Oolong tea	Measurement / ≥ 140 / ≥ 90 mmHg or history of hypertension and currently receiving treatment	1,507	42.2 ± 14.8	711/796
Fatma Celik 2008 (35)	Turkey	Cross-sectional study	FFQ	Black tea	Measurement / ≥ 130 / ≥ 85 mmHg	220/230	48.76 ± 8.10	450/0
Hongxiang Li 2012 (36)	China	Cross-sectional study	Questionnaire	/	Measurement / ≥ 140 / ≥ 90 mmHg or prevalent hypertension	561	52.8 ± 14.2	277/284
Hidenobu Takami 2013 (37)	Japan	Cross-sectional study	Questionnaire	Green tea	Measurement / ≥ 130 / ≥ 85 mmHg or hypertension treatment	554	35–70	409/145
Giuseppe Grosso 2014 (38)	Italy	Cross-sectional study	Questionnaire	/	Measurement / > 130 / > 85 mmHg or treatment of previously diagnosed hypertension	1064/825	50.2 ± 16.3	760/1129
Masoud M MALEKZADEH 2014 (39)	Iran	Cross-sectional study	FFQ	Black tea, Green tea	Measurement / ≥ 140 / ≥ 90 mmHg or history of hypertension and currently receiving treatment	21,350/28,695	40–75	21,234/28,811
Li W 2015 (40)	Singapore	Cross-sectional study	Questionnaire	Black tea, Oolong tea, Green tea	Measurement / ≥ 140 / ≥ 90 mmHg or self-reported history/ treatment for hypertension	565/559	59.82 ± 9.96	469/655
Giuseppe Grosso 2015 (41)	Poland	Cross-sectional study	FFQ	/	Measurement / ≥ 130 / ≥ 85 mmHg or use of hypertensive medication	8,821	45–69	4291/4530
Xiaoyi Zhang 2016 (42)	China	Cross-sectional study	Questionnaire	/	Measurement / ≥ 140 / ≥ 90 mmHg or history of hypertension	17,396/32,036	30–79	20,522/28,910
Jieyun Yin 2017 (43)	China	Cross-sectional study	Questionnaire	Black tea, Oolong tea, Green tea	Measurement / ≥ 140 / ≥ 90 mmHg and (or) use of hypertensive medication	3008/1571	67.63 ± 6.32	2200/2379
Agnieszka Micek 2018 (44)	Poland	Cross-sectional study	Questionnaire(Polish Tables of Food Composition and Nutritional Value)	/	Measurement / ≥ 130 / ≥ 85 mmHg or use of hypertensive medication	5,146	≥ 20	2301/2845
Adefris Chuka 2020 (45)	Ethiopia	Cross-sectional study	Questionnaire	/	Measurement / ≥ 140 / ≥ 90 mmHg and (or) use of hypertensive medication	3,346	44.59 ± 11.17	1674/1672
Agnieszka Micek 2021 (46)	Italy	Cross-sectional study	Questionnaire	/	Measurement /≥ 140 / ≥ 90 mmHg or Self-report or receiving treatment	2044	≥18	/
Xiaodong Peng 2021 (47)	China	Cross-sectional study	Questionnaire	Green tea	Self-reported hypertension / ≥ 140 / ≥ 90 mmHg	9,277	85.45 ± 12.17	3636/5641
Xing-Xuan Dong 2021 (48)	China	Cohort study	Questionnaire	Green tea, other tea	Measurement / ≥ 130 / ≥ 85 mmHg or use of hypertensive medication	3,005	67.3 ± 5.7	1547/1458
Xiao-Ge NIU 2021 (49)	China	Cohort study	Questionnaire	/	Measurement / ≥ 140 / ≥ 90 mmHg and (or) use of hypertensive medication	38,913	49.2 ± 12.3	13,846/25,067
Qiuyi Zhang 2021 (50)	Chian	Cross-sectional study	Questionnaire	Green tea, White tea, Black tea, Yellow tea, other tea	Measurement / ≥ 140 / ≥ 90 mmHg	11,809	60.60 ± 11.93	4776/7033
Yuxiang Yang 2022 (51)	China	Cohort study	FFQ	/	Measurement / ≥ 130 / ≥ 85 mmHg and (or) use of hypertensive medication	43,757	18–59	19,439/24,318
Ying Zhao 2023 (52)	China	Cross-sectional study	Questionnaire	Green tea, Scented tea, Dark tea, Sweet tea, Black tea, Oolong tea, Yellow tea, and White tea.	Measurement / ≥ 140 / ≥ 90 mmHg	76,673	30–79	29,700/46,973
Jing Quan 2023 (53)	China	Cohort study	FFQ	Green tea	Measurement / ≥ 140 / ≥ 90 mmHg	6,633	33–35	0/6633

Participant counts are shown as reported in the original source extraction. In some studies, grouped counts rather than a single total sample size were provided; sex distribution is presented separately when available.

### 3.3 Results of the literature quality assessment

Quality assessment results for the 16 cross-sectional studies are presented in Supplementary Table 1 and those for the 4 cohort studies in Supplementary Table 2. All cross-sectional studies scored ≥ 8 on the AHRQ checklist. NOS scores for cohort studies ranged from 7 to 9. Collectively, the included studies were of relatively high quality, laying a solid foundation for the conclusions of the present meta-analysis.

### 3.4 Results of the meta-analysis

#### 3.4.1 Cross-sectional studies

**Figure 2:**
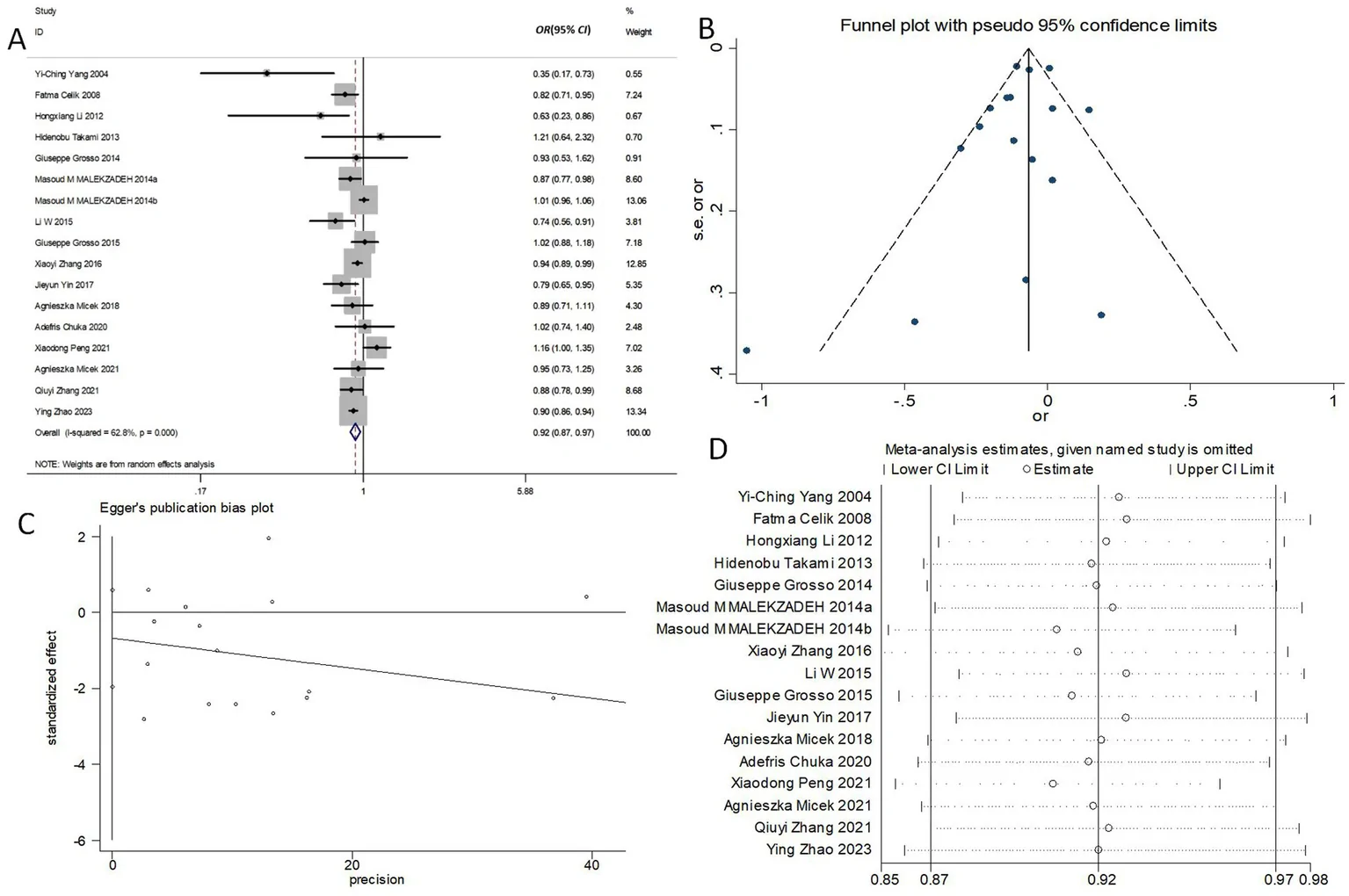
Relationship between tea intake and hypertension based on cross-sectional studies. **(A)** forest plot; **(B)** funnel plot; **(C)** Egger’s test; **(D)** sensitivity analysis.

This meta-analysis included 16 Cross-Sectional Studiesevaluating the association between tea consumption and hypertension. Considerable between-study heterogeneity was observed (*p* < 0.05, *I^2^* = 62.8%); therefore, a random-effects model was used. Tea consumption was associated with a modestly lower likelihood of hypertension, with a pooled relative association estimate of 0.92 (95% *CI*: 0.87–0.97, *Z* = 3.01, *p* < 0.05; Figure 2A). The scatter points in the funnel plot were symmetrically distributed, with only a few studies falling outside the 95% confidence limits. The Egger test result (*p* = 0.269) indicated no significant publication bias (Figures 2B,C). Leave-one-out sensitivity analysis showed no material change in the pooled estimate, supporting the robustness of the overall association (Figure 2D).

#### 3.4.2 Subgroup analysis of cross-sectional studies

**Figure 3:**
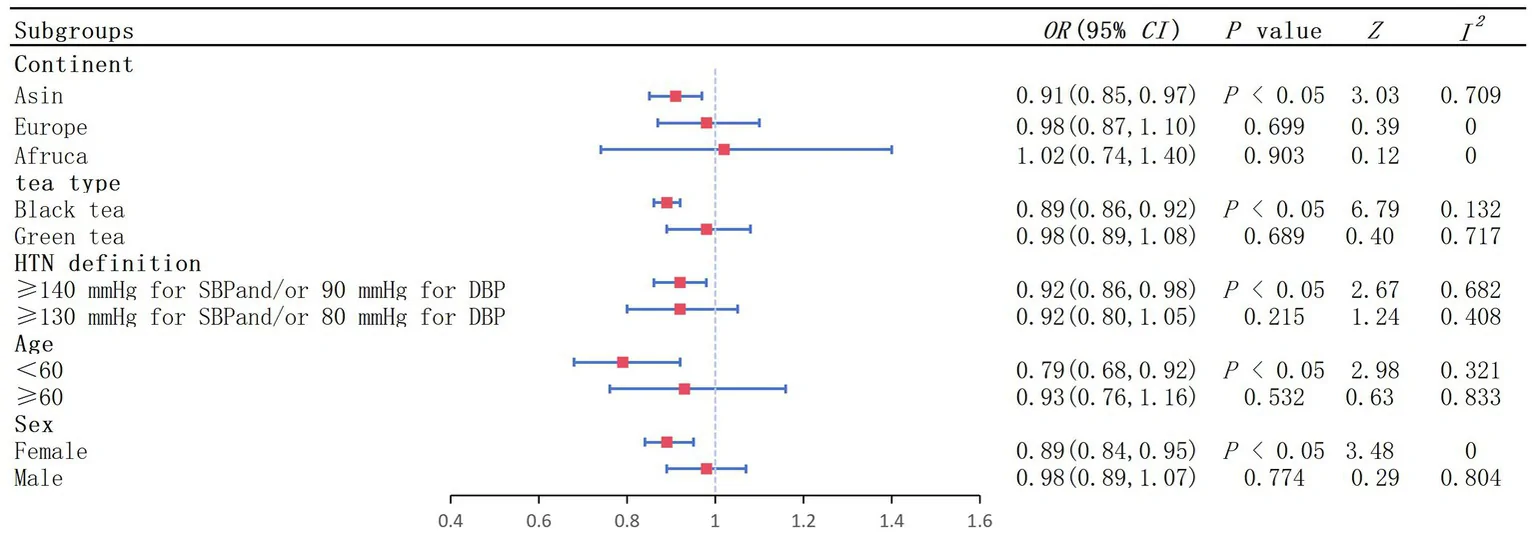
Subgroup analysis of the association between tea intake and hypertension in cross-sectional studies.

To further explore heterogeneity, subgroup analyses limited to cross-sectional studies were performed according to continent, tea type, hypertension diagnostic threshold, age, and sex. Results are summarized in Figure 3 and the corresponding Supplementary figures.

After stratification by continent, heterogeneity differed substantially across regions. Heterogeneity was low in Europe (*I^2^* = 0.0%) and Africa (*I^2^* = 0.0%) but remained high in Asia (*I^2^* = 70.9%); accordingly, a random-effects model was used for Asian studies. A statistically significant inverse association was observed in Asia (*OR* = 0.91, 95% *CI*: 0.85–0.97, *p* < 0.05), whereas no statistically significant association was observed in Europe (0.98, 95% *CI*: 0.87–1.10, *p* = 0.699) or Africa (1.02, 95% *CI*: 0.74–1.40, *p* = 0.903; Supplementary Figure 1). When stratified by tea type, black tea was associated with a lower likelihood of hypertension (0.89, 95% *CI*: 0.86–0.92; *I^2^* = 13.2%), whereas green tea was not statistically significant (0.98, 95% *CI*: 0.89–1.08; *I^2^* = 71.7%; Supplementary Figure 2). Stratification by hypertension diagnostic threshold showed similar inverse associations under thresholds of ≥140/90 mmHg and ≥130/85 mmHg, yet only the subgroup with the cutoff of ≥140/90 mmHg reached statistical significance. (Supplementary Figure 3). Age-stratified analyses suggested a more apparent inverse association among study populations with a mean age < 60 years than among those aged ≥ 60 years (Supplementary Figure 4). Sex-stratified analyses suggested an inverse association in women but not in men (Supplementary Figure 5). However, many subgroups contain few studies and use study-level rather than individual-level characteristics. These should remain explicitly hypothesis-generating, not framed as true effect modification.

#### 3.4.3 Cohort studies

**Figure 4:**
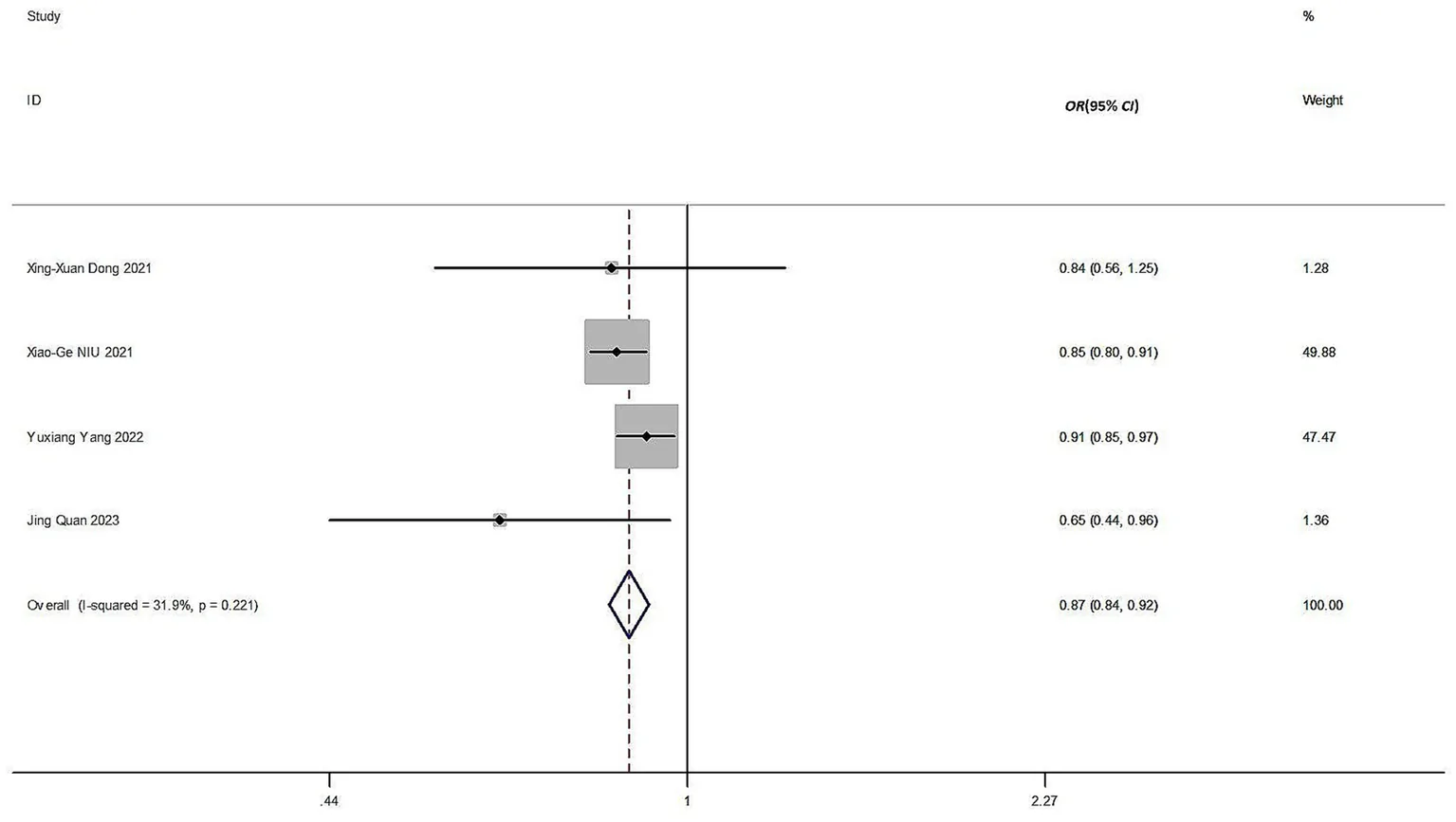
Relationship between tea intake and hypertension based on cohort studies.

Four studies analyzed the relationship between tea consumption and hypertension. The heterogeneity analysis showed low heterogeneity (*I*^2^ = 31.9%, *p* = 0.278), so a fixed-effects model was adopted for pooled analysis. The pooled results demonstrated that tea consumption was significantly associated with a reduced risk of hypertension (*OR* = 0.87, 95% *CI* (0.84–0.92), *Z* = 5.77, *p* < 0.001; Figure 4).

#### 3.4.4 Dose–response analysis

**Figure 5:**
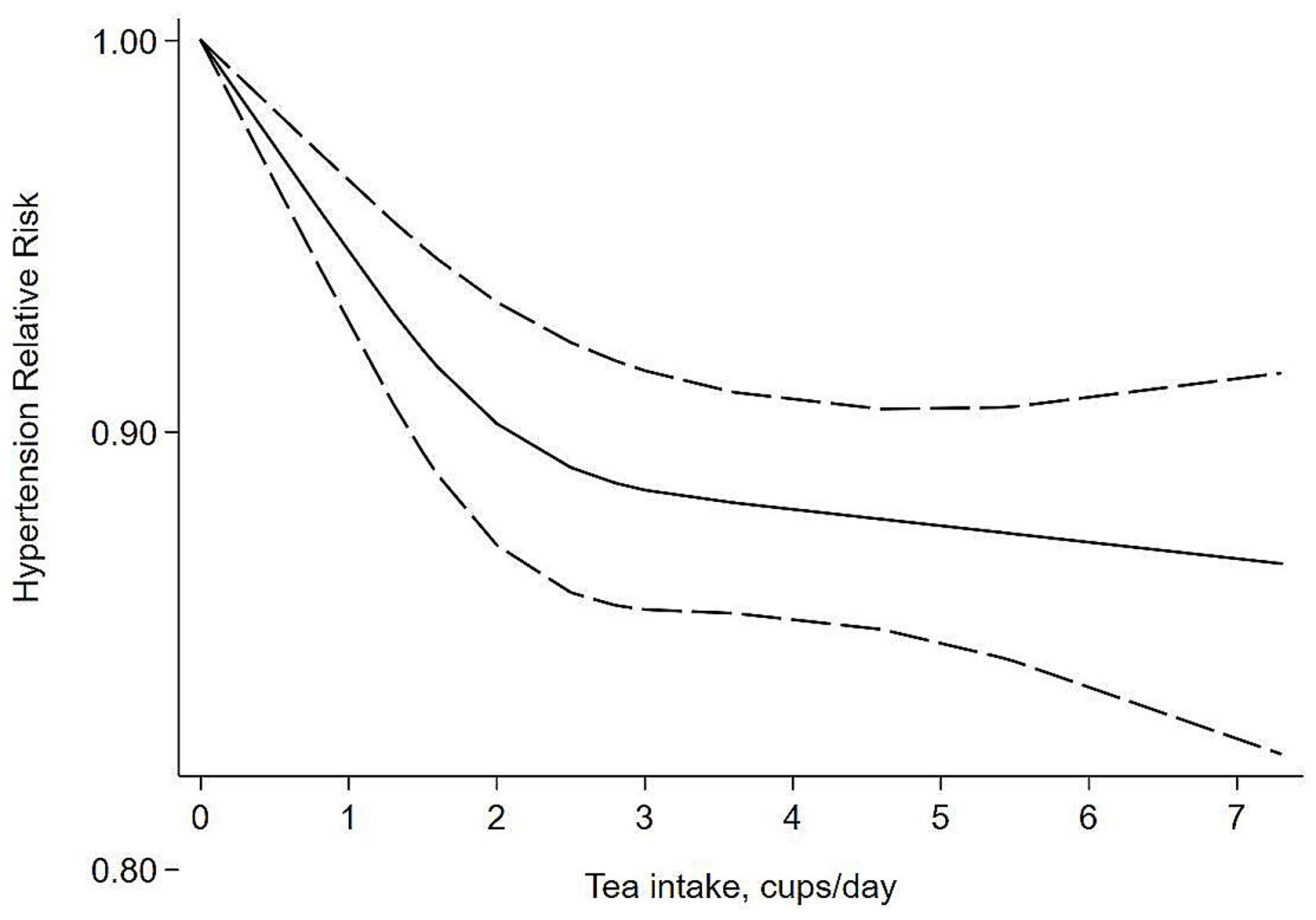
Dose–response analysis of the relationship between tea consumption frequency and hypertension.

Dose–response analysis required at least three exposure categories together with sufficient quantitative information for category-specific risk estimation. After excluding studies that reported only continuous estimates or did not provide adequate category-level data, five studies were included in the dose–response analysis, consisting of three cross-sectional studies and two cohort studies. Restricted cubic spline modeling suggested a significant nonlinear association between tea consumption and hypertension (*P* for nonlinearity < 0.05). The curve indicated an initially steeper inverse pattern at lower reported intake levels, followed by an apparent attenuation or plateau at higher intake levels (Figure 5). Furthermore, sensitivity analyses stratified by study type were conducted to verify the robustness of this nonlinear relationship, with detailed results shown in Supplementary Figure 6. Detailed information of all included studies and dose conversion procedures are summarized in Supplementary Table 3, and these results should therefore be interpreted with caution given the necessary cross-study unit conversion.

## 4 Discussion

This systematic review and meta-analysis synthesized observational evidence on habitual tea consumption and hypertension and found that tea intake was associated with a modestly lower likelihood of hypertension. The magnitude of the pooled association was small, and substantial heterogeneity remained across studies. The present findings therefore support an observational association rather than definitive evidence that tea consumption prevents hypertension. Dose–response analyses suggested a nonlinear pattern, with a steeper inverse association at lower reported intake levels and an apparent plateau at higher levels, but these results should be interpreted cautiously because exposure metrics varied across studies and required harmonization.

Several biologically plausible mechanisms may underlie the observed association. Tea contains catechins, theaflavins, caffeine, and other polyphenols that may influence endothelial nitric oxide signaling, oxidative stress, vascular reactivity, and renin–angiotensin system activity (23–26). Tea consumption might also be linked to body-weight regulation and other lifestyle patterns relevant to blood pressure control (27, 28). However, these mechanisms were not tested directly in the present study, and they should be regarded as biological plausibility rather than mechanistic proof derived from this meta-analysis.

Cross-sectional subgroup analysis revealed differential associations between tea type and hypertension risk: black tea was significantly associated with a reduced risk of hypertension, whereas no statistically significant association was observed for green tea. The tea fermentation process alters the composition of active constituents, which may influence the strength of the association with hypertension. Black tea is rich in theaflavins and thearubigins (29). On one hand, theaflavins can reverse homocysteine-induced endothelial dysfunction in the aorta and alleviate endoplasmic reticulum stress (30). On the other hand, theaflavins and thearubigins scavenge reactive oxygen species, attenuate lipid peroxidation, maintain vascular cell membrane integrity, and inhibit the secretion of pro-inflammatory cytokines to mitigate vascular inflammation (31). Green tea, in contrast, is primarily composed of catechins (29), which confer vascular protective effects by improving oxidative stress status and suppressing inflammatory pathways (32). However, previous studies have demonstrated that green tea extracts exhibit stronger antioxidant activity ex vivo, whereas black tea demonstrates more pronounced effects in *in vivo* models (8, 31). Furthermore, brewing conditions and extraction methods also influence the content and stability of bioactive constituents in tea (33). These factors may collectively affect the observed associations between different tea types and hypertension risk.

The subgroup analyses based on cross-sectional data also warrant careful interpretation. Inverse associations appeared more evident among Asian populations, black tea consumers, women, participants younger than 60 years, and studies with a hypertension cutoff of ≥140/90 mmHg. Nevertheless, these subgroup results may reflect differences in study availability, tea-processing patterns, beverage preparation, exposure measurement, diagnostic thresholds, confounder adjustment, and reporting practices rather than true individual-level effect modification. Accordingly, these findings should be considered hypothesis-generating. The same caution applies to the age and sex analyses, especially where subgroup definitions were based partly on study-level characteristics rather than individual-participant data.

This study has several limitations. First, all included studies were observational; therefore, residual confounding and reverse causation cannot be excluded. Second, exposure assessment was heterogeneous and often based on self-report or questionnaires, introducing possible recall error and misclassification. Third, several studies covered adults aged 18–34 years who could not be excluded; their low hypertension prevalence may dilute the true tea-hypertension association. Fourth, cross-sectional studies cannot establish temporality, and tea consumption may cluster with healthier lifestyle patterns, raising the possibility of healthy-user bias. Fifth, the included studies varied in tea type, strength, brewing method, intake frequency, and adjustment for major confounders such as salt intake, alcohol consumption, smoking, body mass index, physical activity, and socioeconomic status. Finally, relatively few studies were available from non-Asian populations, which limits the generalizability of subgroup comparisons.

In summary, habitual tea consumption was associated with a modestly lower likelihood of hypertension in observational studies, and the dose–response pattern appeared to be nonlinear. These findings may be useful for hypothesis generation and for framing future dietary research, but they should not be interpreted as proof that tea consumption prevents hypertension. Additional high-quality prospective studies with more standardized exposure assessment and, where feasible, carefully designed intervention studies are needed to clarify the magnitude, consistency, and clinical relevance of this association.

## Data Availability

The original contributions presented in the study are included in the article/Supplementary material, further inquiries can be directed to the corresponding author.
