# Supplementary material for: The association between tea consumption and hypertension: a systematic review and meta-analysis
Source: Front Nutr. 2026 Aug 20;13:1883708. doi: 10.3389/fnut.2026.1883708 (PMC13537974; doi:10.3389/fnut.2026.1883708)
Supplement: Supplementary file 1 [file Supplementary_file_1.DOCX]

Supplementary Materials Directory

Supplementary Table 1 Quality of included studies based on the Newcastle–Ottawa scale.	1

Supplementary Table 2 Assessment of the quality of cross-sectional studies based on the criteria recommended by the US Agency for Healthcare Quality and Research (AHRQ).	2

Supplementary Figure 1 Subgroup analysis of the relationship between tea drinking and hypertension was performed by continent-forest map.	3

Supplementary Figure 2 Subgroup analysis of the relationship between tea drinking and hypertension was performed by tea type-forest map.	4

Supplementary Figure 3 The relationship between tea drinking and hypertension was analyzed by subgroup analysis of hypertension diagnostic criteria-forest map.	5

Supplementary Figure 4 Subgroup analysis of the relationship between tea drinking and hypertension was performed by age-forest map.	6

Supplementary Figure 5 Subgroup analysis of the relationship between tea drinking and hypertension was performed by gender-forest map.	7

Supplementary Figure6 Sensitivity analysis of dose–response associations between tea consumption and hypertension risk.	8

Supplementary Table 3 Dose conversion assumptions for unifying tea consumption into cups/day in dose–response analysis.	9

**Supplementary Table 1** Quality of included studies based on the Newcastle–Ottawa scale**.**

|  | Selection (0–4 points) | | | | Comparability  (0–2 points) | Outcome (0–3 points) | | | Total  score |
| --- | --- | --- | --- | --- | --- | --- | --- | --- | --- |
|  | Appropriateness  of case  definition | Representativeness  of cases | Selection  of  controls | Definition  of  controls | Comparability  of cases and  controls | Exposure  assessment | Method of  exposure  assessment | Non- response  Rate |  |
| Xing-Xuan Dong 2021 | 1 | 1 | 1 | 1 | 1 | 1 | 1 | 0 | 7 |
| Xiao-Ge NIU 2021 | 1 | 1 | 1 | 1 | 2 | 1 | 1 | 1 | 9 |
| Yuxiang Yang 2022 | 1 | 1 | 1 | 1 | 2 | 1 | 1 | 0 | 8 |
| Jing Quan 2023 | 1 | 1 | 1 | 1 | 2 | 1 | 1 | 1 | 9 |

**Supplementary Table 2** Assessment of the quality of cross-sectional studies based on the criteria recommended by the US Agency for Healthcare Quality and Research (AHRQ).

| **Included studies （n=16）** | **A** | **B** | **C** | **D** | **E** | **F** | **G** | **H** | **I** | **J** | **K** | **Total score** |
| --- | --- | --- | --- | --- | --- | --- | --- | --- | --- | --- | --- | --- |
| Yi-Ching Yang 2004 | 1 | 1 | 1 | 1 | 0 | 1 | 1 | 1 | 0 | 1 | 1 | 9 |
| Fatma Celik 2008 | 1 | 1 | 1 | 1 | 0 | 1 | 1 | 1 | 0 | 0 | 1 | 8 |
| Hongxiang Li 2012 | 1 | 1 | 1 | 1 | 0 | 1 | 1 | 1 | 0 | 1 | 1 | 9 |
| Hidenobu Takami 2013 | 1 | 1 | 1 | 0 | 0 | 1 | 1 | 1 | 1 | 1 | 1 | 9 |
| Giuseppe Grosso 2014 | 1 | 1 | 1 | 0 | 0 | 1 | 1 | 1 | 0 | 1 | 1 | 8 |
| Masoud M MALEKZADEH 2014 | 1 | 1 | 1 | 1 | 0 | 1 | 1 | 1 | 1 | 1 | 1 | 10 |
| Li W 2015 | 1 | 1 | 1 | 1 | 0 | 1 | 1 | 1 | 0 | 1 | 1 | 8 |
| Giuseppe Grosso 2015 | 1 | 1 | 1 | 1 | 0 | 1 | 1 | 1 | 0 | 1 | 1 | 9 |
| Xiaoyi Zhang 2016 | 1 | 1 | 1 | 1 | 0 | 1 | 0 | 1 | 0 | 1 | 1 | 8 |
| Jieyun Yin 2017 | 1 | 1 | 1 | 1 | 0 | 1 | 1 | 1 | 0 | 1 | 1 | 9 |
| Agnieszka Micek 2018 | 1 | 1 | 1 | 0 | 0 | 1 | 1 | 1 | 0 | 1 | 1 | 8 |
| Adefris Chuka 2020 | 1 | 1 | 1 | 1 | 0 | 1 | 1 | 1 | 0 | 1 | 1 | 9 |
| Agnieszka Micek 2021 | 1 | 1 | 1 | 0 | 0 | 1 | 1 | 1 | 0 | 1 | 1 | 8 |
| Xiaodong Peng 2021 | 1 | 1 | 1 | 1 | 0 | 0 | 1 | 1 | 0 | 1 | 1 | 8 |
| Qiuyi Zhang 2021 | 1 | 1 | 1 | 1 | 0 | 1 | 1 | 1 | 1 | 1 | 1 | 10 |
| Ying Zhao 2023 | 1 | 1 | 1 | 1 | 0 | 1 | 1 | 1 | 0 | 1 | 1 | 9 |
| A:Define the source of information (survey, record review). B:List inclusion and exclusion criteria for exposed and unexposed subjects (cases and controls) or refer to previous publications. C:Indicate time period used for identifying patients. D:Indicate whether or not subjects were consecutive if not population-based. E:Indicate if evaluators of subjective components of study were masked to other aspects of the status of the participants. F:Describe any assessments undertaken for quality assurance purposes (e.g., test/retest of primary outcome measurements). G:Explain any patient exclusions from analysis. H:Describe how confounding was assessed and/or controlled. I:If applicable, explain how missing data were handled in the analysis. J:Summarize patient response rates and completeness of data collection. K:Clarify what follow-up, if any, was expected and the percentage of patients for which incomplete data or follow-up was obtained. | | | | | | | | | | | | |

**Supplementary Figure 1** Subgroup analysis of the relationship between tea drinking and hypertension was performed by continent-forest map.

**Supplementary Figure 2** Subgroup analysis of the relationship between tea drinking and hypertension was performed by tea type-forest map.

**Supplementary Figure 3** The relationship between tea drinking and hypertension was analyzed by subgroup analysis of hypertension diagnostic criteria-forest map.

**Supplementary Figure 4** Subgroup analysis of the relationship between tea drinking and hypertension was performed by age-forest map.

**Supplementary Figure 5** Subgroup analysis of the relationship between tea drinking and hypertension was performed by gender-forest map.

**Supplementary Figure 6** Sensitivity analysis of dose–response associations between tea consumption and hypertension risk.(A) Cross-sectional studies; (B) Cohort studies.

**Supplementary Table 3** Dose conversion assumptions for unifying tea consumption into cups/day in dose–response analysis

| study | author | year | type | Outcome Assessment | Tea Intake Assessment | Type of Tea | Report of Tea Intake | Adjustments | Odds ratio | 95% CI lower | 95% CI upper | dose | cases | peryears |
| --- | --- | --- | --- | --- | --- | --- | --- | --- | --- | --- | --- | --- | --- | --- |
| 1 | Hidenobu Takami | 2013 | cross-sectional study | Measurement / ≥ 130 / ≥ 85 mmHg or hypertension treatment | Questionnaire | Green tea | Tea ＜1, 2-3, and ≥4 cups/day | age, sex, total energy intake,  physical activity, smoking, drinking habits,  and coffee consumption. | 1 | 1 | 1 | 0.4 | 23 | 112 |
|  |  |  |  |  |  |  |  |  | 1.01 | 0.57 | 1.82 | 1.7 | 90 | 400 |
|  |  |  |  |  |  |  |  |  | 1.21 | 0.64 | 2.32 | 4.6 | 59 | 214 |
| 2 | Masoud M MALEKZADEH | 2014 | cross-sectional study | Measurement /  ≥ 140 / ≥ 90 mmHg  or history of hypertension and currently receiving treatment | FFQ | Black tea, Green tea | Tea ＜690, 691-1035, 1036-1500and ＞1500 ml/day | sex, age, BMI, place of residence,  literacy, ethnicity, physical activity, smoking,  black and green tea consumption and wealth score | 1 | 1 | 1 | 1.5 | 6919 | 14,936 |
|  |  |  |  |  |  |  |  |  | 0.92 | 0.88 | 0.97 | 3.6 | 5103 | 11,926 |
|  |  |  |  |  |  |  |  |  | 0.90 | 0.85 | 0.95 | 5.4 | 4874 | 11,758 |
|  |  |  |  |  |  |  |  |  | 0.86 | 0.81 | 0.91 | 7.3 | 4454 | 11,325 |
| 3 | Giuseppe Grosso | 2015 | cross-sectional study | Measurement /  ≥ 130 / ≥ 85 mmHg  or use of hypertensive medication | FFQ | NA | Tea ＜1, 1-2, and ≥3 cups/day | gender, age, educational level, occupational level,  physical activity, smoking status, alcohol drinking,  total energy intake, and tea consumption | 1 | 1 | 1 | 0.5 | 1081 | 1743 |
|  |  |  |  |  |  |  |  |  | 0.90 | 0.80 | 1.01 | 1.6 | 3049 | 5145 |
|  |  |  |  |  |  |  |  |  | 0.78 | 0.69 | 0.88 | 2.6 | 1211 | 1933 |
| 4 | Xiao-Ge NIU | 2021 | cohort study | Measurement /  ≥ 140 / ≥ 90 mmHg  and (or) use of hypertensive medication | Questionnaire | NA | Tea 0, 50, 100and ≥150 g/month | age, sex, body mass index, region, baseline systolic blood pressure,smoking,  drinking, physical activity, education level,  fasting blood glucose, total cholesterol,  and family history of hypertension | 1 | 1 | 1 | 0 | 4357 | 28,799 |
|  |  |  |  |  |  |  |  |  | 0.95 | 0.85 | 1.07 | 0.8 | 317 | 2199 |
|  |  |  |  |  |  |  |  |  | 0.82 | 0.74 | 0.90 | 1.6 | 439 | 3267 |
|  |  |  |  |  |  |  |  |  | 0.83 | 0.76 | 0.91 | 2.8 | 639 | 4465 |
| 5 | Yuxiang Yang | 2022 | cohort study | Measurement /  ≥ 140 / ≥ 90 mmHg  and (or) use of hypertensive medication | FFQ | NA | Tea 0, 1-2, 3-4, and ≥5 cups/day | gender,age,BMI,livingarea,educationlevel,income,marital status,currentsmoker,excessivedrinking,physicalactivity,sleepingduration,sedentary behavior,medicalexaminationwithinoneyear,DASH-dietscore,dailyenergyintake,and family history for hypertension and diabetes | 1 | 1 | 1 | 0 | 14604 | 30,806 |
|  |  |  |  |  |  |  |  |  | 0.93 | 0.87 | 1 | 1.3 | 2213 | 4631 |
|  |  |  |  |  |  |  |  |  | 0.89 | 0.82 | 0.96 | 3 | 1734 | 3434 |
|  |  |  |  |  |  |  |  |  | 0.91 | 0.85 | 0.97 | 4.6 | 2608 | 4891 |
